# Ophthalmological symptoms in children with intracranial cysts

**DOI:** 10.1038/s41598-017-13266-7

**Published:** 2017-10-19

**Authors:** Anna Gotz Wieckowska, Lidia Glowka, Agata Brazert, Marta Pawlak

**Affiliations:** Department of Ophthalmology University of Medical Sciences Dluga ½, Poznan, 61-848 Poland

## Abstract

The purpose of this study was to perform an ophthalmological assessment in children with intracranial cysts and to assess the correlation between the occurrence of cysts and visual disorders. The documentation of 46 children with intracranial cysts, monitored by the Children’s Outpatient Ophthalmology Clinic, Poznan, Poland was analysed. The best corrected visual acuity (BCVA), the alignment of the eyes, visual evoked potentials (VEP), comprehensive eye examination were performed in all patients. Additional ophthalmological tests were conducted to eliminate other causes of decreased visual acuity.Included in the final analysis were 26 children (52 eyes). The average age at the last visit was 10.3 years. Sixteen children (61.5%) had arachnoid cysts located in the posterior cranial fossa, 3 children (11.5%) in the middle cranial fossa, while 7 children (27%) had a pineal cyst. Decreased BCVA was found in 13 children, abnormal VEP in 13, strabismus in 14 patients (53.9%), nystagmus in 5 patients (19.2%), and double vision in 2 patients (7.7%). Numerous visual disorders in children with intracranial cysts suggest the necessity to carry out enhanced ophthalmological diagnostics in these patients. In the examined patient group, visual disorders occurred mostly in the case of arachnoid cysts of the posterior fossa.

## Introduction

While carrying out an ophthalmological assessment of children, we sometimes come across a diagnostic problem while trying to determine what causes decreased visual acuity. In our clinical practice, we have observed a large group of children who, apart from decreased best corrected visual acuity (BCVA) and often abnormal visual evoked potentials (VEP), have been diagnosed with an intracranial arachnoid cyst or a pineal cyst through imaging of the central nervous system (CNS). In these young patients, no causes for this state have been found, despite further ophthalmological assessment.

Arachnoid cysts (ACs) are common. Most recent major studies have estimated arachnoid cyst prevalence by imaging at 2%^1,2^. Arachnoid cysts may be very large or very small, but the size does not correlate precisely with symptoms or the need for treatment^3^. In the available literature, large groups of children with ACs were analysed, where they were evaluated for coexisting neurological disorders. Most authors agree that arachnoid cysts are occasionally present with neurologic signs or symptoms and, in most cases, are asymptomatic and found incidentally. It is universally believed that clinicians should exercise caution when ascribing any non-specific symptom, such as headache, behaviour disturbances, or epilepsy, to the presence of an arachnoid cyst^3^. However, in the analysed groups, the authors do not mention conducting an ophthalmological assessment. Meanwhile, in the literature, one can find numerous descriptions of individual cases in which arachnoid cysts were accompanied by visual disorders^4–11^.

Pineal cysts are a frequent occurrence as well. The prevalence of pineal cysts upon imaging the head in children is 1.9%^12^. However, the clinical significance and management of these cysts is not well defined^13^.

The purpose of this study was to perform an ophthalmological assessment in children with intracranial cysts and to assess the correlation between the occurrence of cysts and visual disorders.

## Methods

The analysis of the medical history of 46 children visiting Children’s Outpatient Ophthalmology Clinic of the Department of Ophthalmology University of Medical Sciences, Poznan, Poland between March 2002 and October 2016 for various reasons, diagnosed with intracranial cysts. Some children visited the clinic with the CNS imaging tests already done for reasons other than ophthalmological, while for the rest it was ordered due to ophthalmological indications.

The criteria for inclusion into the analysed group were: the occurrence of an intracranial cyst, confirmed through imaging and age below 18 years at the first examination. The exclusion criteria: changes other than a cyst in the CNS upon imaging (MRI or CT), lack of cooperation during the ophthalmological tests (BCVA and/or VEP examinations), amblyopia, the occurrence of an eye disease that explained the decreased visual acuity, metabolic diseases, genetic syndromes, the birth age below 32 weeks of pregnancy, the coexistence of arachnoid and pineal cysts, developmental defects of the skull, or an incomplete medical history.

In the ophthalmological assessment of the analysed group of children, the BCVA was evaluated. This means that, for all children, the refraction test was performed after cycloplegia (1% cyclopentolate) and an evaluation of visual acuity was performed with optimal correction. The LCD (Frey) panel was used for the visual acuity assessment. The results were interpreted as: normal or decreased visual acuity relative to the patient’s age, based on the criteria of the American Academy of Ophthalmology^14^.

All children underwent a full ophthalmological assessment, including an evaluation of the alignment of the eyes (the occurrence of a squint or nystagmus) and examination of the anterior segment and eye fundus.

VEPs were performed, according to the International Society for Clinical Electrophysiology of Vision (ISCEV) standards (Retiport System, RolandConsult, Germany), in all but one patient. In the case of one child, the test was not performed due to photosensitive epilepsy of the patient. Pattern-reversal VEPs were performed in children who could cooperate and retain stable fixation during the examination. In children with nystagmus, pattern onset/offset VEPs were performed. Flash VEPs (FVEP) were performed with a hand-held stimulator (Babyflash, RolandConsult, Germany). The shape of the waveform, peak times, and amplitudes in each VEPs result were analysed and the patient was assigned to one of the two groups: normal or abnormal VEP.

In selected cases, in order to rule out structural and functional abnormalities of the retina, an electrophysiological and structural assessment of the retina using electroretinography (ERG) and optical coherence tomography (OCT) was performed.

Visual field (VF) testing was not performed although authors are aware of its importance as a diagnostic tool and its localizing value. The reliable VF results could not be obtained in all patients and it’s statistical analysis would not be suitable for interpretation.

Bioethical Commission was informed about the research conducted. Due to their retrospective nature, absence of the characteristics of a medical experiment as well as non-disclosure of the patients’ personal data, the permission of the Bioethical Commission was not required.

Supporting data are available to Editorial Board Members and referees.

## Results

Based on the inclusion and exclusion criteria, finally 26 children (52 eyes) were included in the analysis: 19 boys (73.1%) and 7 girls (26.9%). Ten children were excluded from the study due to coexisting changes in the CNS (i.e. agenesis of the corpus callosum in 2 children, coexistence of arachnoid cysts and the pineal cysts in 2 children, an underdevelopment of the falx cerebri in 1 child, ischaemic changes of the brain in 3 children, post-traumatic changes of the brain in 1 child, a cyst of the septum pellucidum and vergae ventricle in 1 child). Five children were diagnosed with an eye disease, which explained the decreased BCVA and/or abnormal VEP result (amblyopia in 1 child, bilateral optic nerve atrophy in 2 children without a genetic basis ruled out, stage 4 foveal hypoplasia in 1 child, 1 child with the retinal dystrophy). Three children were excluded due to the lack of cooperation during the ophthalmological assessment, 1 child had craniostenosis and 1 child’s medical history was incomplete.

The period of observation was between one month to 14 years, 3.3 years on average. An average age during the first examination was 7 years (between 2.7 and 15.8 years), and 10.3 years (between 3.8 and 19.5 years) during the last visit.

In the analysed group the CNS imaging for ophthalmological reasons was performed in 16 out of 26 children (61.5%). The indications included: an abnormal optic disc in 3 children (1 optic disc oedema, 1 unilateral optic disc pallor, 1 bilateral increased c/d ratio); a decreased BCVA with normal VEP in 2 children; a decreased BCVA with abnormal VEP in 6 children; nystagmus in 3 children; a double vision in 2 children.

In the case of 10 children (38.5%), the indications for CNS imaging other than ophthalmological were: headaches in 2 children, delayed psychomotor development in 4 children, syncope episodes in 1 child, cranial nerve VII paralysis in 1 child, head trauma in 1 child, epilepsy in 1 child.

Sixteen children had arachnoid cysts of the posterior cranial fossa, in the case of 3 children it was located in the middle cranial fossa, while 7 children had pineal cysts.

A decreased BCVA was found in 13 patients (50%) in 20 eyes (38.5%), a strabismus in 14 patients (53.9%): convergent strabismus in 8 children, divergent strabismus in 6 children; nystagmus in 5 patients (19.2%); a double vision in 2 patients (7.7%).

VEPs were performed in 25 patients; in one patient, the examination was not performed due to photosensitive epilepsy. Abnormal VEP results were present in 13 patients. In the case of 11 children (42.3%), normal visual acuity and VEP were found. In one patient with decreased visual acuity, a normal VEP result was recorded.

In the tested group, apart from ophthalmological symptoms, 6 children (23%) experienced a delayed psychomotor development, 2 (7.7%) suffered from epilepsy, 3 (11.5%) from headaches and other symptoms (Tables 1, 2 and 3).

**Table 1:** The clinical characteristics of 16 children with posterior cranial fossa arachnoid cyst included in the study.

**Case**	**Gender**	**Age at last control (years)**	**BCVA RE/LE**	**VEP RE/LE**	**Ocular symptoms**	**Neurological symptoms**
1	male	10.6	N/N	AN/AN	XT	
2	male	6.0	D/D	N/AN	ET	
3	male	13.5	D/D	AN/AN	ET	DPD, E
4	male	5.8	D/N	AN/N	ET	DPD, ADHD
5	female	7.4	D/D	Ø	XT, N	DPD, E
6	male	7.0	D/N	AN/N	ET	
7	male	7.8	N/D	N/AN	ET	
8	female	3.8	N/N	N/N	XT	IFNP
9	male	5.3	N/N	AN/AN	XT, AHP	DPD
10	female	18.7	D/D	AN/AN	N, AHP, FH, UP	
11	male	10.9	N/N	N/N	ECVD, EPE	SE
12	male	17.8	N/N	N/N	BOH	
13	male	15.2	D/N	AN/N		AD
14	male	7.3	N/N	N/N	ECVD	H
15	male	12.9	D/D	N/N	FH	DPD
16	male	6.8	D/N	AN/N		DPD

Abbreviations: BCVA RE/LE- best corrected visual acuity right eye/left eye; BCVA: D- decreased N- norm; VEP RE/LE- visual evoked potentials right eye/left eye; VEP: AN – abnormal, N- normal; ET- esotropia; XT- exotropia; N- nystagmus; FH- foveal hypoplasia (I° OCT); AHP- abnormal head posture; UP- unilateral ptosis (clinically insignificant); ECVD- episodes of color vision disturbance; EPE- eye pain episodes; BOH- bilateral optic disc oedema history; DPD- delayed psychomotor development; E- epilepsy; H- headache; SE- syncope episodes; AD- adjustment disorder; ADHD- attention deficit hyperactivity disorder; IFNP- infectious facial nerve palsy.

**Table 2:** The clinical characteristics of 3 children with middle cranial fossa arachnoid cyst included in the study.

**Case**	**Gender**	**Age at last control (years)**	**BCVA RE/LE**	**VEP RE/LE**	**Ocular symptoms**	**Neurological symptoms**
1	male	15.9	N/D	N/AN	N, UONA	
2	male	6.1	N/N	N/N	ET	
3	male	13.3	N/N	N/N		

Abbreviations: BCVA RE/LE- best corrected visual acuity right eye/left eye; BCVA: D- decreased N- norm; VEP RE/LE- visual evoked potentials right eye/left eye; VEP: AN – abnormal, N- normal; ET- esotropia; N- nystagmus; UONA- unilateral optic nerve atrophy.

**Table 3:** The clinical characteristics of 7 children with pineal cyst included in the study.

**Case**	**Gender**	**Age at last control (years)**	**BCVA RE/LE**	**VEP RE/LE**	**Ocular symptoms**	**Neurological symptoms**
1	female	4.3	N/N	N/N	XT	
2	male	11.9	N/N	N/N	XT	H
3	female	19.5	N/N	N/N	ET, DV	
4	female	8.8	N/N	N/N	DV	
5	male	5.8	N/N	N/N	N	V
6	male	8.9	D/D	N/AN	BONDE	
7	female	16.7	D/D	AN/AN	ET, N	H

Abbreviations: BCVA RE/LE- best corrected visual acuity right eye/left eye; BCVA: D- decreased N- norm; VEP RE/LE- visual evoked potentials right eye/left eye; VEP: AN – abnormal, N- normal; ET- esotropia; XT- exotropia; N- nystagmus; DV- double vision; BONDE- bilateral optic nerve disc elevation; H- headache; V- vertigo.

## Discussion

Arachnoid cysts are fluid-filled malformations of the arachnoid tissue. Congenital, genetic, and traumatic factors have been suggested as the underlying mechanisms^2^. The location of cysts varies greatly. Based on the research carried out by a number of authors, these cysts are most often located in the middle cranial fossa (1.15). In the tested group: in 61.5% of children arachnoid cysts were found in the posterior cranial fossa; in 11.5% of children - in the middle cranial fossa. In 26.9% of children pineal cyst was diagnosed.

Many authors have identified a greater prevalence of arachnoid cysts in males^15–17^. Similarly, in our study the majority of the analysed group were boys (73.1%).

Pineal cysts are frequently discovered on brain MRIs in children^3^. Pineal cyst prevalence changes with age, but these cysts are found more frequently in girls than in boys in all age groups^3,13,18^. Although generally considered asymptomatic, these cysts have been occasionally associated with neurological sequelae including headaches, hydrocephalus, extraocular movement abnormalities, and Parinaud syndrome^13^. In our group, 7 children had pineal cysts, and girls represented 57.1% of that group. In this group, two children had double vision and two children had nystagmus.

It is difficult to prove a direct correlation between a decreased visual acuity and the occurrence of an intracranial cyst. For the purpose of this work, while selecting the examined group through establishing the inclusion and exclusion criteria, we strived to eliminate all patients diagnosed with other disorders which might have had an impact on visual acuity. Particular emphasis should be placed on amblyopia, which is defined as unilateral or, less commonly, bilateral reduction of best-corrected visual acuity that occurs in the setting of an otherwise normal eye, or a structural abnormality involving the eye or visual pathway, with reduction in visual acuity that cannot be attributed only to the effect of the structural abnormality^14^.

Performing both BCVA and VEP testing makes it possible to differentiate between amblyopia and other causes of a decreased BCVA. A decreased BCVA was found in 13 (50%) of our patients, in 21 (38.5%) eyes.

There are many publications in the literature on the subject of intracranial ACs. In studies dealing with ACs, in a study of 488 children between 0–14 years of age with arachnoid cysts, Huang *et al*. mentions only two children with visual disorders, but it is not clearly stated in the work whether all the children had undergone ophthalmological evaluation, nor does it specify the disorders^15^. In the work of Al-Holou *et al*.^1^, analysing a group of 309 children with ACs, there is no mention an ophthalmological assessment of their patients, either. The study by Tan *et al*. presented a classification of ACs using magnetic resonance cisternography (MRC) as well as the result of treatment of 23 children, but does not report any visual disorders in any of those cases; just like the other studies mentioned above, there is no information on an ophthalmological assessment^19^.

Meanwhile, in the literature, one can find many descriptions of individual cases of patients with arachnoid cysts, accompanied by visual disorders^5–7,9,10^. Ocular manifestations such as optic disc oedema, optic nerve hypoplasia, nystagmus, and/or oculomotor palsy may be present^20^. In our examined group of children, the most common symptom was a strabismus, occurring in 14 (53.9%) patients. Additionally, 5 (19.2%) patients had nystagmus and 2 (7.7%) had double vision.

The importance of the VEP test in the diagnosis of visual pathway dysfunctions has been emphasised by some authors^4,8,11^. VEPs are visually evoked electrophysiological signals recorded over the visual cortex. The standard protocol is intended for the assessment of prechiasmal function. However, as the visual cortex is activated primarily by the central visual field, VEPs depend on functional integrity of central vision at any level of the visual pathway, thus providing diagnostic information on the functional integrity of the visual system^21^. In this work, the functional ophthalmological assessment was conducted by evaluating BCVA and testing VEP. In the group of our patients, abnormal VEP was found in 13 patients (52%), in 21 eyes (42%): in 10 patients out of 16 with a cyst in the posterior cranial fossa (62.5%), in 1 out of 3 with a cyst in the middle cranial fossa (33.3%) and in 2 out of 7 with a pineal cyst (28.6%).

Published reports indicate that arachnoid cysts may also affect cranial nerves in the posterior fossa, causing symptoms such as vertigo/dizziness and hearing loss or facial palsy. According to some authors, these symptoms are most likely caused by direct pressure of the cyst on the cranial nerves or stretching of the nerves^2^.

The limitations of our work are the small size of the group of patients, the fact that they were referred to an ophthalmologist or made appointments due to visual disorders, as well as the absence of a control group. Further ophthalmological tests of large groups of patients are necessary, particularly in those with a cyst located in the posterior cranial fossa, in order to definitively confirm or rule out the impact of ACs occurring in this area of the visual pathway. The influence of pineal cysts on the sight organ also requires clarification. However, to our knowledge, this is the first study in which an ophthalmological assessment was performed in children with intracranial cysts. Numerous visual disorders in children with intracranial cysts suggest the necessity of performing enhanced ophthalmological diagnostics in these patients. In the examined patient group, visual disorders occurred mostly in the case of arachnoid cysts in the posterior cranial fossa. The ophthalmological assessment in children with intracranial cysts seems to be indispensable.
